# IL-10 and IL-2R as combined predictors of intravenous immunoglobulin resistance in Kawasaki disease: a retrospective cohort study

**DOI:** 10.3389/fimmu.2025.1646502

**Published:** 2025-12-03

**Authors:** Hui-Ying Wang, Sun Chen, Chun Zhang, Ji-Hong Huang

**Affiliations:** 1 Department of Pediatric Cardiac Center, Xinhua Hospital, Shanghai Jiao Tong University School of Medicine, Shanghai, China; 2 Department of Pharmacy, Xinhua Hospital, Shanghai Jiao Tong University School of Medicine, Shanghai, China

**Keywords:** Kawasaki disease, inflammation, cytokines, interleukin-10, interleukin-2 receptor, intravenous immunoglobulin resistance

## Abstract

**Objective:**

Intravenous immunoglobulin (IVIG) resistance in Kawasaki disease (KD) increases the risk of coronary artery lesions (CALs) and the need for additional therapies. Early identification remains a clinical challenge. This study evaluated the predictive value of interleukin (IL)-10 and IL-2 receptor (IL-2R) in detecting IVIG resistance.

**Methods:**

We retrospectively analyzed 529 children with KD treated at Xinhua Hospital, Shanghai Jiao Tong University School of Medicine, from November 2019 to December 2024. Demographic and clinical characteristics, and laboratory data were compared between IVIG-responsive and IVIG-resistant groups. Multivariable logistic regression was used to identify independent predictors, and receiver operating characteristic (ROC) curves were used to assess predictive performance.

**Results:**

Among 529 patients, 88 (16.6%) were IVIG-resistant and 441 (83.3%) were IVIG-responsive. Compared with IVIG-responsive patients, IVIG-resistant patients had significantly higher levels of IL-10 and IL-2R. Both remained independent predictors after adjustment for confounders. ROC analysis demonstrated high predictive accuracy for IL-2R (AUC = 0.825) and limited predictive value for IL-10 (AUC = 0.767). The combination of IL-10 and IL-2R moderately improved predictive accuracy, achieving a better balance between sensitivity and specificity (AUC = 0.834, sensitivity 77.10%, specificity 79.56%). Subgroup analysis revealed that IL-2R had limited predictive value in infants younger than 12 months. However, in patients aged 12 months or older, both IL-10 and IL-2R were significant risk factors for IVIG resistance.

**Conclusion:**

IL-2R is an independent predictor of IVIG resistance in KD in the age group more than 12 months, and IL-10 serves as a complementary marker, and their combined use slightly enhances predictive utility across most age groups.

## 1 Introduction

Kawasaki disease (KD) is an acute systemic vasculitis that predominantly affects children under five years of age and remains the leading cause of acquired heart disease in developed countries (1). Coronary artery lesions (CALs), including dilatation, aneurysms, and stenosis, represent the most severe complications and are associated with long-term cardiovascular morbidity (2). IVIG is the standard first-line therapy, reducing the incidence of CALs from 15-25% to 3-5% (3). Nevertheless, 10-20% of patients fail to respond to IVIG, and these non-responders face a substantially increased risk of severe CALs and require additional treatments such as corticosteroids or biologics (4–9). Thus, reliable early prediction of IVIG resistance is critical to optimize management strategies and prevent adverse outcomes.

Current prediction of IVIG resistance largely relies on clinical parameters, but these models have limited specificity and inconsistent performance across populations due to heterogeneity in biomarkers and patient characteristics (4). Consequently, there is an urgent need to identify novel immunological markers that more directly reflect the underlying pathophysiology of KD.

Given that cytokines control vascular inflammation and immunological activation, recent studies highlight their role in the pathogenesis of KD, especially IL-10 and IL-2R (10, 11). The anti-inflammatory cytokine IL-10 strongly correlates with the severity of some inflammatory disorders, whereas IL-2R indicates T-cell activation and might be a sign of hyperimmune reactions (12, 13). According to earlier research, IL-2R and IL-10 both showed improved sensitivity and diagnostic precision when used as biomarkers to assess inflammatory activity in KD (14). Nevertheless, current predictive models have not integrated these cytokines, and their applicability for individualized prediction of IVIG resistance remains limited due to heterogeneity in clinical parameters, genetic background, and environmental influences (15–17).

One of the immunological hallmarks of KD is systemic T-cell hyperactivation, involving both CD4^+^ helper and CD8^+^ cytotoxic T-cell subsets (18, 19). Elevated IL-10 levels may cause moderate immune paralysis by suppressing antigen-presenting cells such as dendritic cells and macrophages, thereby diminishing the immunomodulatory efficacy of IVIG (20). Concurrently, IL-2R upregulation disrupts the Th1/Th2 balance and impairs CD4^+^ memory T-cell homeostasis, contributing to immune dysregulation (21). During the acute phase of KD, sustained IL-2R release may perpetuate T-cell activation, promote vascular endothelial injury, and ultimately increase the risk of CALs (22).

While previous studies have linked these cytokines to inflammation in KD, their efficacy in predicting IVIG resistance remains unclear. Therefore, this study aimed to systematically evaluate the predictive performance of IL-10 and IL-2R, both individually and in combination, for IVIG resistance in a KD cohort. Through analysis of clinical and laboratory parameters from 529 patients, we sought to establish independent predictive value, thereby providing a scientific basis for implementing targeted therapeutic strategies in high-risk patients.

## 2 Materials and methods

### 2.1 Study population

**Figure 1:**
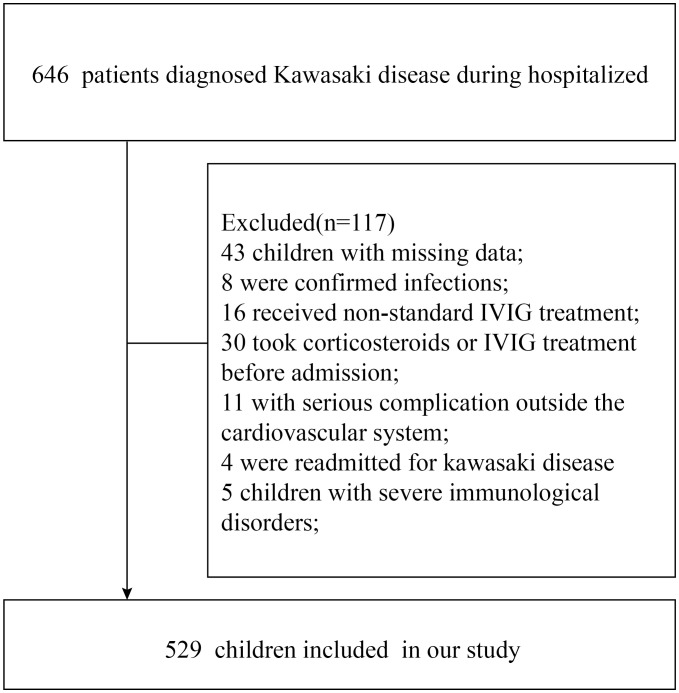
Numbers of study-eligible Kawasaki disease patients.

We conducted a retrospective cohort study including 529 patients with KD who were admitted to the Pediatric Department, Xinhua Hospital, Shanghai Jiao Tong University School of Medicine, between November 2019 and December 2024. The diagnosis of KD was established according to the American Heart Association (AHA) criteria (23). The detailed process of patient screening and selection is illustrated in **Figure 1**. All clinical data and biological samples were collected before December 2024, while data cleaning and statistical analyses were completed by April 2025.

The study protocol was reviewed and approved by the Ethics Committee of Xinhua Hospital, Shanghai Jiao Tong University School of Medicine (Approval No. XHEC-D-2025-093).

### 2.2 Inclusion and exclusion criteria

The diagnosis of classic KD is based on the presence of ≥5 days of fever (first calendar day of fever is illness day 1) and the presence of ≥4 of the 5 principal clinical features (23): 1) erythema and cracking of lips, strawberry tongue, and/or erythema of oral and pharyngeal mucosa; 2) bilateral bulbar conjunctival injection without exudate; 3) rash: maculopapular, diffuse erythroderma, or erythema multiforme-like; 4) erythema and edema of the hands and feet in acute phase and/or periungual desquamation in subacute phase; 5) Cervical lymphadenopathy (≥1.5 cm diameter), usually unilateral.

The diagnosis of incomplete (sometimes referred to as atypical) KD should be considered in any infant or child with prolonged unexplained fever, fewer than 4 of the principal clinical findings, and compatible laboratory or echocardiographic findings (23).

Children were classified into two groups: IVIG-responsive KD, defined as defervescence without recurrence after the initial IVIG infusion, and IVIG-resistant KD, defined as persistent or recurrent fever (≥38 °C) occurring at least 36 hours after completion of the first IVIG infusion (23).

Children were excluded if they met any of the following criteria: (1) Children with missing data during treatment; (2) Children diagnosed with infection upon enrolment; (3) Children who were given non-standard IVIG treatment; (4) children who had received IVIG or corticosteroid treatment prior to treatment; (5) children with serious complications outside the cardiovascular system; (6)children were readmitted for Kawasaki disease; (7)children with severe immunological disorders.

During the acute phase, IVIG was administered to all enrolled patients at a total dose of 2 g/kg, combined with oral aspirin at 30–50 mg/kg/day. After becoming afebrile for the period of 48 to 72 hours, the daily dosage of aspirin was reduced to 3–5 mg/kg. Patients who showed signs of IVIG resistance were given methylprednisolone (2 mg/kg/day) and an extra IVIG infusion (single dose of 2 g/kg).

### 2.3 Data collection

All enrolled patients’ medical records were retrospectively reviewed, including demographic data such as age (months), gender, weight, days of fever before admission, clinical characteristics, IVIG response, and laboratory parameters before IVIG therapy.

C-reactive protein (CRP), white blood cell count (WBC), Neutrophil%, Lymphocyte%, absolute lymphocyte count (ALC), hemoglobin (HB), platelet count (PLT), alanine aminotransferase (ALT), aspartate aminotransferase (AST), sodium (Na), total protein (TP), albumin (ALB), interleukin-8 (IL-8), interleukin-1B (IL-1B), interleukin-6 (IL-6), IL-10, tumor necrosis factor-α (TNF-α), and IL-2R were the laboratory parameters.

Serum cytokine concentrations (IL-6, IL-8, IL-10, IL-1β, TNF-α, and IL-2R) were determined using an electrochemiluminescence (ECL) immunoassay on the Cobas e 601 analyzer (Roche Diagnostics, Basel, Switzerland). Venous blood samples for cytokine measurement were collected at the time of hospital admission, before administration of any medication (including IVIG and aspirin), and during febrile episodes (≥38.0 C).

### 2.4 Statistical analyses

Categorical variables were expressed as counts and percentages, and group differences were compared using the χ² test. Continuous variables were presented as the mean ± standard deviation or median (25th–75th percentile), and independent sample T test or Mann–Whitney U test was used to compare the differences between the two groups depending on the data distribution. Variables with *p* < 0.05 in the univariate analysis were further included in a multivariate logistic regression analysis to identify independent risk factors for IVIG-resistant KD. Multivariable logistic regression models were constructed, with adjustments made for age, sex, and weight as potential confounding variables. In addition, subgroup analyses were performed by clinically established age groups (e.g., <12 months, 12–60 months, >60 months). The variance inflation factor (VIF) was used to assess multicollinearity, and logistic regression (univariate and multivariate) analyses were performed to identify risk variables. The predictive performance of each biomarker was assessed using receiver operating characteristic (ROC) curve analysis, and the area under the curve (AUC) was calculated. Optimal cutoff values were determined based on the Youden index. All statistical tests were two-sided, and a *p* < 0.05 was considered statistically significant. Analyses were performed using SPSS version 26.0 (IBM Corp., Armonk, NY, USA).

## 3 Results

### 3.1 Baseline characteristics

Between November 2019 and December 2024, a total of 646 KD patients had been treated at the pediatric department of our institution. Based on the exclusion criteria, 117 cases were excluded. Our analysis ultimately comprised 529 KD patients (**Figure 1**), 88 of whom (16.6%) were identified as IVIG-resistant.

Baseline demographic and clinical characteristics of the IVIG-responsive (55.1% male) and IVIG-resistant groups (60.2% male) are summarized in **Table 1**. Compared with the responsive group, IVIG-resistant patients showed a significantly higher incidence of CALs (*p* < 0.05). Laboratory values are presented in **Table 2**. IVIG-resistant patients showed significantly lower levels of ALC, PLT, TP, ALB and Na, while CRP, Neutrophil%, ALT, IL-6, IL-8, IL-10, TNF-α, and IL-2R levels were significantly elevated (all *p* < 0.05).

**Table 1:** Baseline demographic and clinical characteristics in IVIG-resistant patients and IVIG-responsive patients.

Variables	IVIG-resistant (n=88)	IVIG-responsive (n=441)	*p*
Demographic characteristics			
male, n(%)	53(60.2)	243(55.1)	0.377
Weight(kg), median (IQR)	14.28(11.00, 18.00)	14.00(11.00, 18.40)	0.933
Age in months, median (IQR)	33.28(17.61, 56.03)	33.42(18.48, 55.34)	0.875
Clinical characteristics			
Days from fever onset to IVIG initiation, median (IQR)	5.00(5.00, 6.00)	5.00(5.00, 6.00)	0.207
Fever, n (%)	88(100)	441(100)	1.000
Cervical lymphadenitis, n (%)	71(80.7)	358(81.2)	0.913
Conjunctival injection, n (%)	83(94.3)	408(92.5)	0.550
Oral mucosal changes, n (%)	48(54.5)	285(64.6)	0.074
Skin rash, n (%)	71(80.7)	317(71.9)	0.088
Extremity changes, n (%)	37(42.0)	187(42.4)	0.950
Complete KD, n (%)	53(60.2)	286(64.9)	0.409
CALs, n (%)	37(42.0)	48(10.9)	<0.001*

*p<0.05.

CALs, coronary artery lesions.

**Table 2:** Laboratory values in IVIG-resistant patients and IVIG-responsive patients.

Variables	IVIG-resistant patients (n=88)	IVIG-responsive patients (n=441)	*p*
CRP(mg/L), median (IQR)	79.00(49.00, 153.75)	66.00(37.00, 97.50)	0.002*
WBC(×10^9^/L), median (IQR)	12.85(8.93, 16.29)	13.04(9.99, 16.14)	0.569
Neutrophil%, median (IQR)	74.10(65.03, 84.30)	66.20(55.70, 77.00)	<0.001*
Lymphocyte%, median (IQR)	16.85(9.73, 26.10)	24.25(15.50, 33.48)	<0.001*
ALC(×10^9^/L), median (IQR)	1.66(1.12, 3.08)	2.86(1.93, 4.32)	<0.001*
HB(g/L), mean ± SD	108.60 ± 9.83	110.04± 9.498	0.199
PLT(×10^9^/L), median (IQR)	308.00(251.00, 404.75)	352.50(274.00, 429.25)	0.006*
ALT(U/L), median (IQR)	32.00(18.00, 80.50)	22.50(14.00, 55.73)	0.004*
AST(U/L), median (IQR)	34.50(25.00, 50.25)	32.00(25.00, 44.63)	0.590
TP(g/L), median (IQR)	61.15(56.95, 65.45)	64.05(60.00, 68.68)	<0.001*
ALB(g/L), median (IQR)	36.15(32.70, 38.13)	37.50(35.10, 40.20)	<0.001*
Na(mmol/L), median (IQR)	135.30(133.20, 137.10)	136.80(135.08, 138.40)	<0.001*
IL-8(pg/mL), median (IQR)	43.50(22.30, 108.50)	23.40(13.00, 77.25)	0.001*
IL-1B(pg/mL), median (IQR)	17.65(9.94, 35.48)	15.45(6.98, 27.33)	0.218
IL-6(pg/mL), median (IQR)	84.30(32.00, 224.25)	40.95(17.48, 92.35)	<0.001*
IL-10(x10 pg/mL), median (IQR)	3.73(1.48, 8.37)	1.22(0.59, 2.37)	<0.001*
TNF-α(pg/mL), median (IQR)	29.05(22.65, 39.08)	21.50(16.03, 29.45)	<0.001*
IL-2R(x10^2^ U/mL), median (IQR)	32.34(24.79, 45.98)	16.23(11.89, 23.41)	<0.001*

*p<0.05.

CRP, C-reactive protein; WBC, white blood cell count; Neutrophil%, Neutrophil Percentage; Lymphocyte%, Lymphocyte percentage; ALC, absolute lymphocyte count; HB, hemoglobin; PLT, platelet count; ALT, alanine aminotransferase; AST, aspartate aminotransferase; TP, Total Protein; ALB, Albumin; Na, sodium;IL-8, interleukin-8; IL-1B, interleukin-1B; IL-6, interleukin-6; IL-10, interleukin-10; TNF-α, tumor necrosis factor-α; IL-2R, interleukin-2 receptor.

### 3.2 Multivariate logistic regression analysis results

To identify independent risk factors for IVIG-resistant KD, fourteen variables (ALC, PLT, TP, ALB, Na, CRP, Neutrophil%, ALT, IL-6, IL-8, IL-10, TNF-α, IL-2R and CALs levels) were included in a multivariate logistic regression analysis. After adjusting for confounding factors such as age, gender, and weight, the multivariate analysis indicated that CALs, IL-10, and IL-2R were significant independent predictors of IVIG-resistant KD (*p*<0.05). Multicollinearity analysis confirmed that the variance inflation factor (VIF) for all variables was below 5, indicating no significant multicollinearity among the variables (**Supplementary Table S1**). The results showed that CALs, IL-10 and IL-2R levels were independent predictors of IVIG resistance in children with KD (**Table 3**).

**Table 3:** Results of logistic regression analyses of IVIG resistance in KD patients.

Variables	B	SE	OR (95%CI)	*P*
Sex	0.387	0.322	1.472(0.783, 2.768)	0.230
Age in months	0.005	0.014	1.005(0.977, 1.033)	0.774
Weight(kg)	-0.002	0.063	0.998(0.883, 1.128)	0.973
CALs	2.173	0.356	8.787(4.373, 17.656)	<0.001*
CRP(mg/L)	-0.004	0.004	0.996(0.989, 1.003)	0.217
Neutrophil%	0.012	0.017	1.012 (0.979, 1.046)	0.475
ALC(×10^9^/L)	-0.312	0.171	0.732(0.524, 1.022)	0.067
PLT(×10^9^/L)	0.001	0.001	1.001(0.998, 1.004)	0.618
ALT(U/L)	0.000	0.002	1.000(0.996, 1.004)	0.894
Na(mmol/L)	-0.065	0.057	0.937(0.838, 1.049)	0.260
TP(g/L)	-0.051	0.031	0.950(0.893, 1, 010)	0.100
ALB(g/L)	0.057	0.057	1.058(0.947, 1.183)	0.317
IL-8(pg/mL)	0.000	0.000	1.000(1.000, 1.000)	0.722
IL-6(pg/mL)	0.000	0.001	1.000(0.999, 1.002)	0.685
IL-10(x10 pg/mL)	0.128	0.052	1.137(1.027, 1.258)	0.013*
TNF-α(pg/mL)	-0.002	0.003	0.998(0.993, 1.003)	0.472
IL-2R(x10^2^U/mL)	0.063	0.012	1.065(1.041, 1.090)	<0.001*

*p<0.05.

CALs, coronary artery lesions; CRP, C-reactive protein; Neutrophil%, Neutrophil Percentage; ALC, absolute lymphocyte count; PLT, platelet count; ALT, alanine aminotransferase; Na, sodium; TP, Total Protein; ALB, Albumin; IL-8, interleukin-8; IL-6, interleukin-6; IL-10, interleukin-10; TNF-α, tumor necrosis factor-α; IL-2R, interleukin-2 receptor.

We performed multivariate logistic regression analysis to assess the association of IL-10 and IL-2R levels with IVIG resistance across different age subgroups. Subgroup analysis revealed that IL-10 was an independent risk factor for IVIG resistance across all age groups: < 12 months (OR = 1.169, 95% CI: 1.002-1.363, P = 0.0466), 12–60 months (OR = 1.333, 95% CI: 1.208-1.471, P < 0.0001), and > 60 months (OR = 1.415, 95% CI: 1.164-1.719, P = 0.0005). IL-2R served as a significant risk factor for IVIG resistance in the 12–60 months (OR = 1.121, 95% CI: 1.087-1.155, P < 0.0001) and > 60 months (OR = 1.106, 95% CI: 1.055-1.159, P < 0.0001) age groups but was not statistically significant in the < 12 months age group (OR = 1.026, 95% CI: 0.989-1.065, P = 0.1660) (**Table 4**).

**Table 4:** Multivariate logistic regression analysis of IVIG resistance risk by age groups.

Index	IL-10 OR (95%CI) P-value	IL-2R OR (95%CI) P-value
< 12 months	1.169 (1.002, 1.363) 0.0466	1.026 (0.989, 1.065) 0.1660
12–60 months	1.333 (1.208, 1.471) <0.0001	1.121 (1.087, 1.155) <0.0001
> 60 months	1.415 (1.164, 1.719) 0.0005	1.106 (1.055, 1.159) <0.0001

IL-10, interleukin-10; IL-2R, interleukin-2 receptor.

### 3.3 The predictive values for IVIG-resistant KD

**Figure 2:**
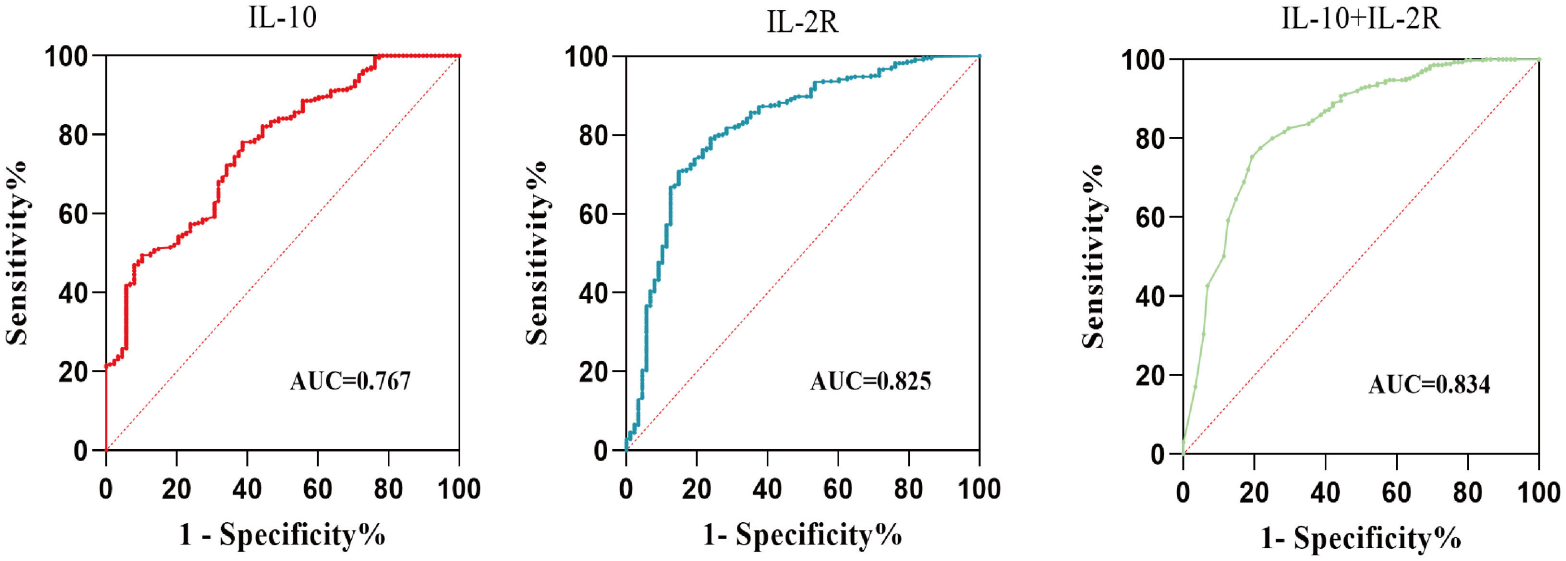
The ROC curve analysis for the prediction of IVIG resistance in KD patients.

ROC analysis was used to evaluate the predictive values of IL-10, IL-2R, and the combination of IL-10 and IL-2R for IVIG resistance in children with KD. ROC analysis of the entire cohort showed that IL-10 alone had limited predictive value (AUC = 0.767, sensitivity 78.23%, specificity 61.36%), while IL-2R demonstrated high discriminative power (AUC = 0.825, sensitivity 70.98%, specificity 85.23%). The combination of IL-10 and IL-2R slightly enhanced the predictive efficiency, leading to a balanced improvement in sensitivity while maintaining good specificity (AUC = 0.834, sensitivity 77.10%, specificity 79.56%) (**Figure 2**, **Table 5**).

**Table 5:** The predictive values for IVIG-resistant KD.

Index	AUC	P	95%CI	Sensitivity(%)	Specificity(%)	Maximum value of the Youden index	Cut-off level
IL-10	0.767	<0.0001*	(0.715, 0.819)	78.23	61.36	0.396	2.695 (x10 pg/mL)
IL-2R	0.825	<0.0001*	(0.774, 0.876)	70.98	85.23	0.562	21.84 (x10^2^ U/mL)
IL-10+IL-2R	0.834	<0.0001*	(0.784, 0.883)	77.10	79.56	0.567	0.13

*p<0.05.

IL-10, interleukin-10; IL-2R, interleukin-2 receptor.

## 4 Discussion

In this study, we demonstrated that IL-2R is a predictor of IVIG resistance in KD, with high specificity and consistent performance in the age group more than 12 months. IL-10 serves as a complementary marker, adding modest value in subgroup analyses but with limited incremental benefit in the combined model. These findings highlight the central role of immune dysregulation in IVIG resistance and suggest that biomarker-based risk stratification may provide a more precise approach for identifying high-risk patients (10, 19, 24).

### 4.1 IL-2R as a marker of immune activation

IL-2R exhibited strong discriminative power (AUC = 0.825) and clinical utility. Its high specificity (85.23%) minimizes false positives, reducing unnecessary aggressive treatment in low-risk patients. IL-2R, composed of α, β, and γ chains, is essential for T-lymphocyte activation and proliferation. Elevated IL-2R levels have been associated with a variety of immune-mediated disorders, as they reflect heightened immune cell activation (25, 26). One of the hallmarks of KD is the immune system’s activation, namely that of T cells (13, 20). Our results show that higher IL-2R levels at admission are significantly associated with IVIG resistance, suggesting that elevated immune cell activation may contribute to IVIG resistance. Our findings are consistent with earlier reports linking abnormal T-cell activation to IVIG resistance and adverse outcomes (14), thereby reinforcing IL-2R as a mechanistically plausible and clinically valuable predictor.

### 4.2 The complementary role of IL-10

IL-10 was an independent predictor but with modest clinical impact (OR = 1.137), meaning a 1 pg/mL increase in IL-10 is associated with a 13.7% increased risk of IVIG resistance. Consistent with subgroup analysis results, IL-10 serves as a reliable predictor in infants under 12 months, where IL-2R shows limited predictive value. Its higher sensitivity (78.23%) complements IL-2R’s specificity, making it useful for identifying at-risk patients in younger cohorts (<12 months) where IL-2R’s performance is less consistent.

In contrast to the T-cell activation marker IL-2R, we also investigated the anti-inflammatory cytokine IL-10. IL-10 is a key anti-inflammatory cytokine that regulates the immune response by inhibiting the synthesis of pro-inflammatory cytokines (27). It is believed that a major contributing factor to vasculitis in KD is an imbalance of pro-inflammatory and anti-inflammatory cytokines (28). Some investigations have indicated elevated IL-10 levels in KD patients, suggesting its role in the illness process (18, 29). Although the odds ratio for IL-10 was modest (AUC = 0.767), its combination with IL-2R significantly enhanced predictive accuracy. Our research shows that IVIG resistance is linked to greater IL-10 levels upon admission, suggesting a dysregulated anti-inflammatory response in non-responders.

These findings support the hypothesis that IVIG resistance is partially driven by aberrant immune responses (30). IL-10 deficiency has been shown to significantly influence antibody sialylation patterns, particularly through the upregulation of α2, 3-linked sialylation, which may interfere with the anti-inflammatory effects of IVIG therapy (31, 32). Notably, this IL-10-mediated disruption of antibody sialylation may reduce the ability of IVIG to modulate the immune response, thereby contributing to IVIG resistance in KD patients.

### 4.3 Combined model’s marginal benefit

Complementing the pro-inflammatory signal captured by IL-2R, the anti-inflammatory cytokine IL-10 also demonstrated independent predictive value, albeit with a different performance profile. Although the absolute increase in AUC was modest, the combined model achieved a more favorable balance between sensitivity and specificity, which might be clinically preferable in some settings where missing a potential non-responder is of greater concern.

### 4.4 Comparison with other cytokines

Other cytokines, including IL-6, IL-8, and TNF-α, were elevated in resistant patients but did not retain independent predictive significance. This suggests that although these mediators contribute to the inflammatory milieu of KD, their predictive value is less specific than IL-2R and IL-10. Prior studies have reported inconsistent associations for IL-6 and TNF-α, further supporting the need to focus on cytokines with stronger mechanistic relevance and reproducibility across populations.

### 4.5 Clinical implications

The identification of IL-2R and IL-10 as independent predictors of IVIG resistance has important translational potential. The cutoff values determined in this study (IL-2R ≥ 21.84 U/mL, IL-10 ≥ 2.695 pg/mL) provide clinically applicable thresholds that could guide early intervention. Patients exceeding these thresholds may benefit from prompt consideration of adjunctive therapies, such as corticosteroids or biologics, before the development of severe coronary complications. Integrating cytokine biomarkers into existing risk scores may also improve predictive accuracy, addressing the limited applicability of current models across diverse populations.

### 4.6 Limitations and future directions

Several limitations should be acknowledged. First, this was a single-center retrospective study, and the relatively small number of IVIG-resistant cases (n=88) may limit generalizability. Second, cytokine levels were measured only once at admission, preventing assessment of their temporal dynamics during disease progression or in response to therapy. Third, although key confounders such as age, sex, and weight were adjusted for, other demographic and clinical variables may still influence biomarker performance. Finally, because the cohort was derived from a single Chinese center with uniform treatment protocols, validation in multi-center, ethnically diverse populations under different clinical practices is essential. Given the variations in demographics and second-line treatment strategies across regions (e.g., North America and Japan), further validation in diverse clinical settings is warranted.

Future research should therefore focus on prospective, large-scale validation of IL-2R and IL-10 as predictive biomarkers in pre-IVIG risk stratification frameworks. Serial cytokine measurements at predefined intervals (e.g., pre-IVIG, 24 h, 48 h post-treatment) may also provide insights into the dynamic immune responses underlying resistance. In addition, translational studies exploring how modulation of IL-10 and IL-2R pathways may enhance IVIG efficacy could open new therapeutic avenues. Such studies could identify critical therapeutic window and offer greater insights into the underlying pathological processes.

## 5 Conclusion

IL-2R is a clinically useful predictor of IVIG resistance in KD, with consistent performance across most age groups. IL-10 serves as a complementary marker, particularly in younger patients. Their combined use offers modest incremental benefit, supporting personalized risk stratification and targeted therapy. However, further extensive, multi-center research is required to validate these results and investigate the predictive value of these biomarkers in clinical practice.

## Data Availability

The raw data supporting the conclusions of this article will be made available by the authors, without undue reservation.
