# Supplementary material for: IL-10 and IL-2R as combined predictors of intravenous immunoglobulin resistance in Kawasaki disease: a retrospective cohort study
Source: Front Immunol. 2025 Dec 3;16:1646502. doi: 10.3389/fimmu.2025.1646502 (PMC12708526; doi:10.3389/fimmu.2025.1646502)
Supplement: Supplementary file 1 [file Table1.docx]

Supplementary Material

**Supplementary Table 1 Univariate and multivariate logistic analysis for predictors of IVIG resistance before and after IVIG**

| **Characteristics** | | **Univariable** | |  | | **Multivariable** |  |  | | Adjust# |  |  | **VIF** |
| --- | --- | --- | --- | --- | --- | --- | --- | --- | --- | --- | --- | --- | --- |
|  |  | **odds ratio (95%CI)** | ***P*–value** |  | | **odds ratio (95%CI)** | ***P*–value** |  | | **odds ratio (95%CI) *P*–value** | |  |  |
| CALs | 5.940(3.536-9.977) | | <0.001 |  | 8.205(4.129-16.306) | | <0.001 |  | 8.787(4.373-17.656) | | <0.001 |  | 1.061 |
| CPR(mg/L) | 1.007(1.003-1.011) | | <0.001 |  | 0.996(0.989-1.002) | | 0.196 |  | 0.996(0.989-1.003) | | 0.217 |  | 1.433 |
| Neutrophil%, | 1.047(1.028-1.067) | | <0.001 |  | 1.015(0.982-1.049) | | 0.380 |  | 1.012(0.979-1.046) | | 0.475 |  | 2.082 |
| ALC(*109/L) | 0.649(0.543-0.776) | | <0.001 |  | 0.708(0.513-0.977) | | 0.036 |  | 0.732(0.524-1.022) | | 0.067 |  | 1.848 |
| PLT(*109/L) | 0.997(0.995-0.998) | | 0.008 |  | 1.001(0.998-1.004) | | 0.671 |  | 1.001(0.998-1.004) | | 0.618 |  | 1.274 |
| ALT(U/L) | 1.004(1.001-1.007) | | 0.005 |  | 1.000(0.996-1.004) | | 0.889 |  | 1.000(0.996-1.004) | | 0.894 |  | 1.129 |
| Na(mmol/L) | 0.851(0.788-0.918) | | <0.001 |  | 0.951(0.850-1.063) | | 0.374 |  | 0.937(0.838-1.049) | | 0.260 |  | 1.339 |
| TP(g/L) | 0.922(0.890-0.950) | | <0.001 |  | 0.953(0.900-1.010) | | 0.102 |  | 0.950(0.893-1.010) | | 0.100 |  | 1.964 |
| ALB(g/L) | 0.889(0.839-0.943) | | <0.001 |  | 1.052(0.946-1.170) | | 0.350 |  | 1.058(0.947-1.183 ) | | 0.317 |  | 2.029 |
| IL-8(pg/mL) | 1.000(1.000-1.000) | | 0.216 |  | 1.000(1.000-1.000) | | 0.729 |  | 1.000(1.000-1.000) | | 0.722 |  | 1.411 |
| IL-6(pg/mL) | 1.002(1.001-1.003) | | 0.002 |  | 1.000(0.999-1.002) | | 0.684 |  | 1.000(0.999-1.002) | | 0.685 |  | 1.584 |
| IL-10(x10 pg/mL) | 1.306(1.210-1.409) | | <0.001 |  | 1.132(1.025-1.250) | | 0.014 |  | 1.137(1.027-1.258) | | 0.013 |  | 1.382 |
| TNF-α(pg/mL) | 1.001(0.998-1.005) | | 0.477 |  | 0.998(0.993-1.003) | | 0.489 |  | 0.998(0.993-1.003) | | 0.472 |  | 1.228 |
| IL-2R(x100 U/mL) | 1.008(1.006-1.010) | | <0.001 |  | 1.006(1.004-1.008) | | <0.001 |  | 1.006(1.004-1.009) | | <0.001 |  | 1.443 |
| Adjust#: Adjusted odds ratio calculated using multivariable logistic regression analysis, adjusting for age, gender, and weight.  Abbreviations: CALs, coronary artery lesions;CRP, C-reactive protein; Neutrophil%, Neutrophil Percentage; ALC, absolute lymphocyte count; PLT, platelet count; ALT, alanine aminotransferase; Na, sodium; TP, Total Protein; ALB, Albumin;;IL-8, interleukin-8; IL-6,interleukin-6; IL-10, interleukin-10; TNF-α, tumor necrosis factor-α; IL-2R, interleukin-2 receptor. | | | | | | | | | | | | | |
